# Trends in Current Electronic Cigarette Use Among Youths by Age, Sex, and Race and Ethnicity

**DOI:** 10.1001/jamanetworkopen.2023.54872

**Published:** 2024-02-05

**Authors:** Delvon T. Mattingly, Joy L. Hart

**Affiliations:** 1 Center for Health Equity Transformation, College of Medicine, University of Kentucky, Lexington; 2 Department of Behavioral Science, College of Medicine, University of Kentucky, Lexington; 3 Department of Communication, College of Arts and Sciences, University of Louisville, Louisville, Kentucky; 4 Christina Lee Brown Envirome Institute, School of Medicine, University of Louisville, Louisville, Kentucky

## Abstract

This cross-sectional study examines prevalence of electronic cigarette use among youths by age, sex, and race and ethnicity from 2013 to 2022.

## Introduction

Electronic cigarettes (e-cigarettes) are the most used tobacco products among youths in the US^1^ and may facilitate progression into use of more harmful products, such as cigarettes,^2^ which are associated with premature morbidity and mortality.^3^ Although previous research has characterized youth e-cigarette prevalence,^1^ updated descriptive statistics on use trends by key sociodemographic characteristics are needed.^4^

## Methods

This cross-sectional study was deemed exempt from review and informed consent by the University of Kentucky’s institutional review board because it used publicly available, deidentified data in accordance with the Common Rule; the study followed the STROBE reporting guideline. We used data from the National Youth Tobacco Survey (NYTS), an annual, repeated cross-sectional survey of US middle and high school students, from 2013 to 2022.^5^ The total number of respondents across 10 years was 199 113, and we included 186 555 youths with complete information on e-cigarette use, age, sex, and race and ethnicity each year.

Survey delivery mode differed starting in 2021 due to the COVID-19 pandemic.^5^ The NYTS was delivered online and permitted students to take the survey anywhere (eg, at home) as opposed to in school. Comparing estimates from 2021 to 2022 with those of previous years is discouraged and may introduce information bias.^6^ Thus, we created trend breaks and examined trends between 2013 to 2020 and 2021 to 2022. No trends between 2020 and 2021 should be inferred. While NYTS 2021 and 2022 surveys were administered online, methodological differences limit comparisons (eg, changes may be due to methods, behavior, or both).^5^

Current e-cigarette use was defined as past 30-day use. We included self-reported age (9-12, 13-15, and 16-18 years), sex (male and female), and race and ethnicity (Hispanic, non-Hispanic Black, non-Hispanic White, non-Hispanic multiracial, and another race or ethnicity), using categories defined by NYTS investigators, as sociodemographic characteristics. The non-Hispanic multiracial classification included youths who identified as multiple races. Another race or ethnicity included non-Hispanic American Indian or Alaskan Native, non-Hispanic Asian, and non-Hispanic Native Hawaiian or Other Pacific Islander.

We calculated yearly weighted e-cigarette use prevalence overall and by age, sex, and race and ethnicity and assessed changes over time (2013-2020 and 2021-2022) by examining CI overlap (2-sided; significance level of .05). We used Stata statistical software version 16.1 (StataCorp) for data analysis.

## Results

We report demographic and use characteristics by year among 186 555 youths (44.82% aged 13-15 years; 50.65% males; 12.68% non-Hispanic Black, 24.44% Hispanic, 51.75% non-Hispanic White, and 5.68% another race or ethnicity) in the Table. The overall e-cigarette use prevalence was 3.10% in 2013 and peaked at 20.18% in 2019. Between 2021 and 2022, prevalence remained stable, at 7.50% and 9.44%, respectively (Figure). E-cigarette use was consistently more prevalent among older than younger youths. Prevalence among older youths (ages 16-18 years) peaked at 30.01% in 2019 and remained stable from 2021 to 2022.

**Table.** Weighted Prevalence of Current Electronic Cigarette Use

Group	Youths, % (95% CI) (N = 186 555)^a^									
	2013 (n = 16 746)	2014 (n = 20 245)	2015 (n = 16 467)	2016 (n = 19 125)	2017 (n = 16 586)	2018 (n = 18 654)	2019 (n = 18 267)	2020 (n = 14 025)	2021 (n = 19 385)	2022 (n = 27 055)
Overall	3.10 (2.66-3.61)	9.33 (7.98-10.88)	11.28 (10.09-12.59)	8.29 (7.43-9.23)	7.93 (6.69-9.37)	13.86 (12.41-15.45)	20.18 (18.60-21.85)	13.25 (11.42-15.33)	7.50 (6.40-8.77)	9.44 (8.03-11.08)
Age, y^b^										
9-12	0.58 (0.36-0.95)	2.26 (1.73-2.94)	3.30 (2.56-4.25)	2.70 (2.10-3.46)	2.46 (1.82-3.30)	2.82 (2.23-3.56)	5.81 (4.74-7.12)	2.63 (1.90-3.61)	1.87 (1.39-2.52)	2.06 (1.43-2.97)
13-15	2.40 (1.97-2.92)	7.70 (6.45-9.16)	9.27 (8.20-10.47)	7.03 (6.26-7.87)	6.53 (5.59-7.62)	10.60 (9.42-11.90)	18.14 (16.59-19.79	10.67 (9.01-12.59)	5.22 (4.35-6.25)	6.82 (5.69-8.15)
16-18	5.23 (4.32-6.32)	14.88 (12.43-17.70)	17.85 (15.70-20.24)	12.82 (11.19-14.65)	12.78 (10.39-15.62)	23.66 (21.18-26.33)	30.08 (27.61-32.67)	22.73 (19.99-25.73)	13.49 (11.40-15.90)	16.96 (14.96-19.16)
Sex^b^										
Male	3.82 (3.18-4.58)	10.49 (8.89-12.34)	13.23 (11.62-15.02)	9.61 (8.58-10.75)	9.04 (7.67-10.64)	14.85 (13.31-16.52)	20.14 (18.41-21.98)	13.56 (11.63-15.74)	6.99 (5.85-8.34)	8.39 (6.83-10.27)
Female	2.36 (1.94-2.88)	8.17 (6.96-9.57)	9.28 (8.19-10.51)	6.96 (5.89-8.20)	6.79 (5.56-8.26)	12.88 (11.40-14.52)	20.22 (18.47-22.09)	12.94 (10.96-15.20)	8.05 (6.81-9.49)	10.53 (8.90-12.41)
Race and ethnicity^b^										
Hispanic	3.72 (3.03-4.55)	10.94 (9.05-13.18)	12.62 (11.22-14.17)	8.18 (6.84-9.76)	7.31 (5.53-9.61)	10.81 (9.34-12.48)	18.64 (16.82-20.60)	13.69 (11.02-16.87)	5.78 (4.60-7.22)	8.51 (7.38-9.80)
Non-Hispanic Black	1.96 (1.35-2.86)	4.87 (3.75-6.30)	6.41 (5.45-7.53)	5.03 (4.23-5.97)	3.50 (2.65-4.62)	5.24 (4.09-6.68)	13.32 (11.09-15.91)	5.65 (4.27-7.45)	4.23 (2.97-5.97)	8.04 (6.03-10.66)
Non-Hispanic White	3.12 (2.51-3.88)	10.01 (8.21-12.14)	11.54 (10.00-13.29)	9.37 (8.22-10.65)	9.63 (8.14-11.36)	17.99 (16.09-20.07)	23.37 (21.28-25.60)	15.44 (13.32-17.84)	9.47 (7.97-11.21)	10.96 (8.94-13.37)
Non-Hispanic multiracial^c^	3.62 (2.36-5.53)	10.00 (6.85-14.38)	15.77 (11.72-20.89)	9.01 (7.14-11.31)	8.42 (6.17-11.39)	11.66 (9.04-14.91)	18.32 (15.63-21.36)	14.61 (10.61-19.79)	9.45 (7.21-12.31)	10.57 (8.21-13.50)
Another race or ethnicity^d^	2.87 (1.74-4.72)	6.69 (4.91-9.05)	11.26 (6.33-19.26)	4.64 (3.11-6.86)	3.62 (2.25-5.76)	9.50 (7.30-12.28)	13.95 (11.15-17.31)	8.10 (5.28-12.21)	4.06 (2.58-6.31)	3.22 (1.89-5.43)

^a^ Given survey administration (ie, in person to online) and methodological differences in 2021 due to the COVID-19 pandemic, comparing estimates between 2021 and any prior year is discouraged. While surveys were administered solely online, additional methodological differences were introduced for the National Youth Tobacco Survey 2022, limiting comparisons between 2021 and 2022.

^b^ Age, sex, and race and ethnicity were reported to characterize disparities in electronic cigarette use.

^c^ Multiracial refers to youths who identified as multiple races.

^d^ Another race or ethnicity included youths who identified as non-Hispanic American Indian or Alaskan Native, non-Hispanic Asian, and non-Hispanic Native Hawaiian or Other Pacific Islander.

**Figure.**
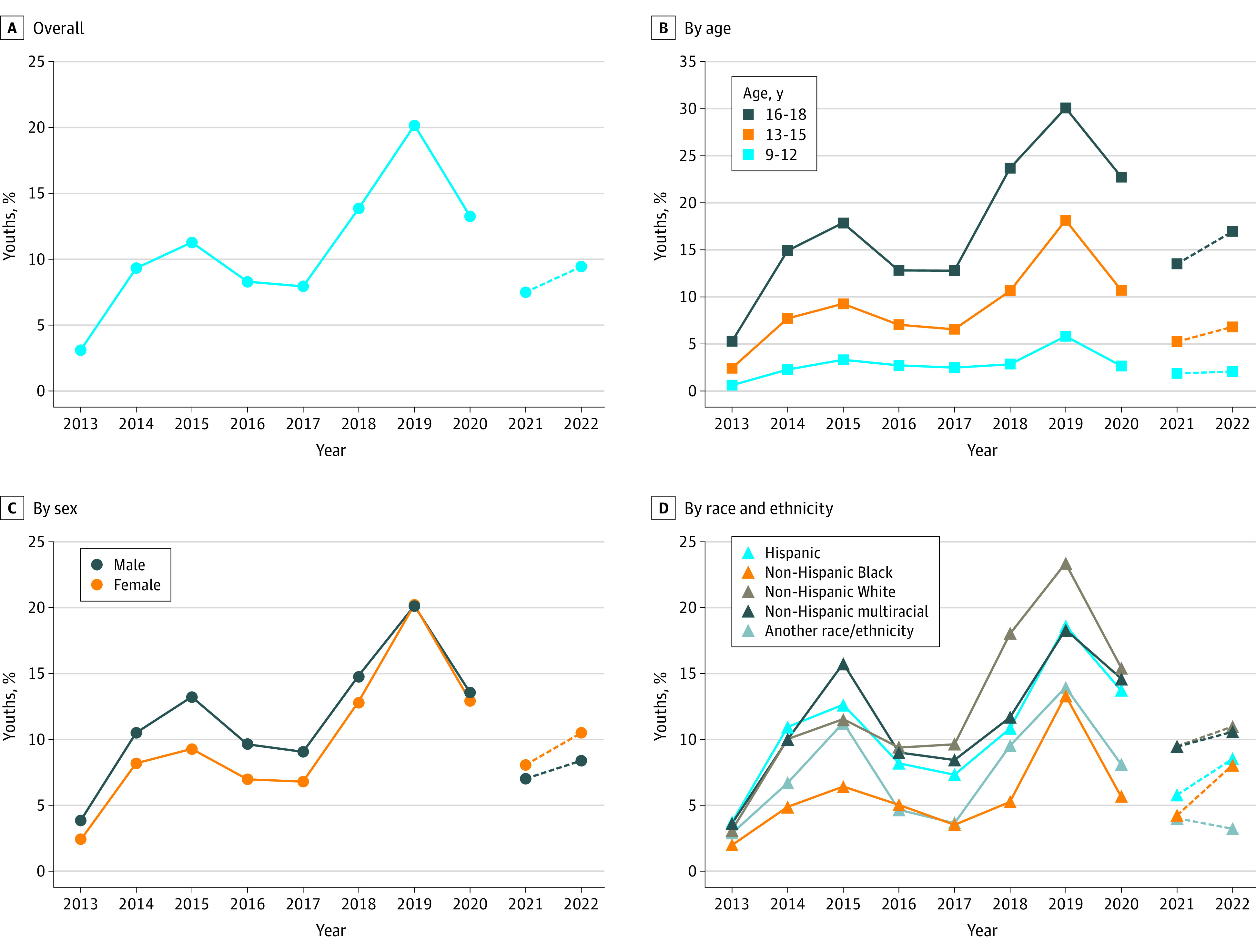
Prevalence of Current Electronic Cigarette Use Trends in prevalence are given overall (A) and by age (B), sex (C), and race and ethnicity (D). Dashed lines (2021-2022) indicate that methodological differences limit trend comparisons.

No differences between use trends by sex were observed until 2015, when males (13.23%) had a higher prevalence than females (9.28%). By 2019, prevalence peaked and was approximately even between males (20.14%) and females (20.22%). In 2021 and 2022, females (8.05% and 10.53%, respectively) had higher use prevalence than males (6.99% and 8.39%, respectively), although this difference was not statistically significant.

Hispanic, non-Hispanic White, and non-Hispanic multiracial youths generally had higher e-cigarette use prevalence from 2013 to 2020 compared with non-Hispanic Black youths. For example, use prevalence in 2019 was high among Hispanic (18.64%) and non-Hispanic White (23.37%) compared with non-Hispanic Black (13.32%) youths. From 2021 to 2022, prevalence increased among Hispanic (5.78%-8.51%) and non-Hispanic Black (4.23%-8.04%) youths.

## Discussion

This cross-sectional study found a substantial increase in youth e-cigarette use prevalence in the early 2010s, peaking in 2019 and declining during the early 2020s to still-concerning levels. From 2021 to 2022, use increased for certain groups, such as Hispanic and non-Hispanic Black youths. Temporal e-cigarette use trends by age, sex, and race and ethnicity highlight disparate groups that require tobacco prevention services. Our study limitations include potential concerns about recall bias and the inability to examine trends between 2020 and 2021.

Overall, these findings update the literature on youth e-cigarette use trends in the US and complement previous research detailing disparities in e-cigarette use by providing a temporal component.^1^ Future research will benefit from examining trends using additional measures of e-cigarette use, such as frequency and flavorings.

## Notes

Supplement.
Data Sharing Statement
