# Supplementary material for: Trends in Current Electronic Cigarette Use Among Youths by Age, Sex, and Race and Ethnicity
Source: JAMA Netw Open. 2024 Feb 5;7(2):e2354872. doi: 10.1001/jamanetworkopen.2023.54872 (PMC10844997; doi:10.1001/jamanetworkopen.2023.54872)
Supplement: Supplement. — Data Sharing Statement [file jamanetwopen-e2354872-s001.pdf]

Data Sharing Statement

Mattingly. Trends in Current Electronic Cigarette Use Among Youths by Age, Sex, and Race and Ethnicity. *JAMA Netw Open*. Published online February 5, 2024.
doi:10.1001/jamanetworkopen.2023.54872

Data
Data available: No

Additional Information
Explanation for why data not available: The data are publicly available at the CDC (https://www.cdc.gov/tobacco/data_statistics/surveys/nyts/index.htm)
